# Supplementary material for: Manipulating dehydrogenation kinetics through dual-doping Co3N electrode enables highly efficient hydrazine oxidation assisting self-powered H2 production
Source: Nat Commun. 2020 Apr 15;11:1853. doi: 10.1038/s41467-020-15563-8 (PMC7160107; doi:10.1038/s41467-020-15563-8)
Supplement: Supplementary file 1 — Supplementary Materials [file 41467_2020_15563_MOESM1_ESM.pdf]

Supplementary Materials

Manipulating Dehydrogenation Kinetics through Dual-Doping Co_3N Electrode Enables Highly-Efficient Hydrazine Oxidation Assisting Self-Powered H_2 Production

Liu et al.

Supplementary Figures:

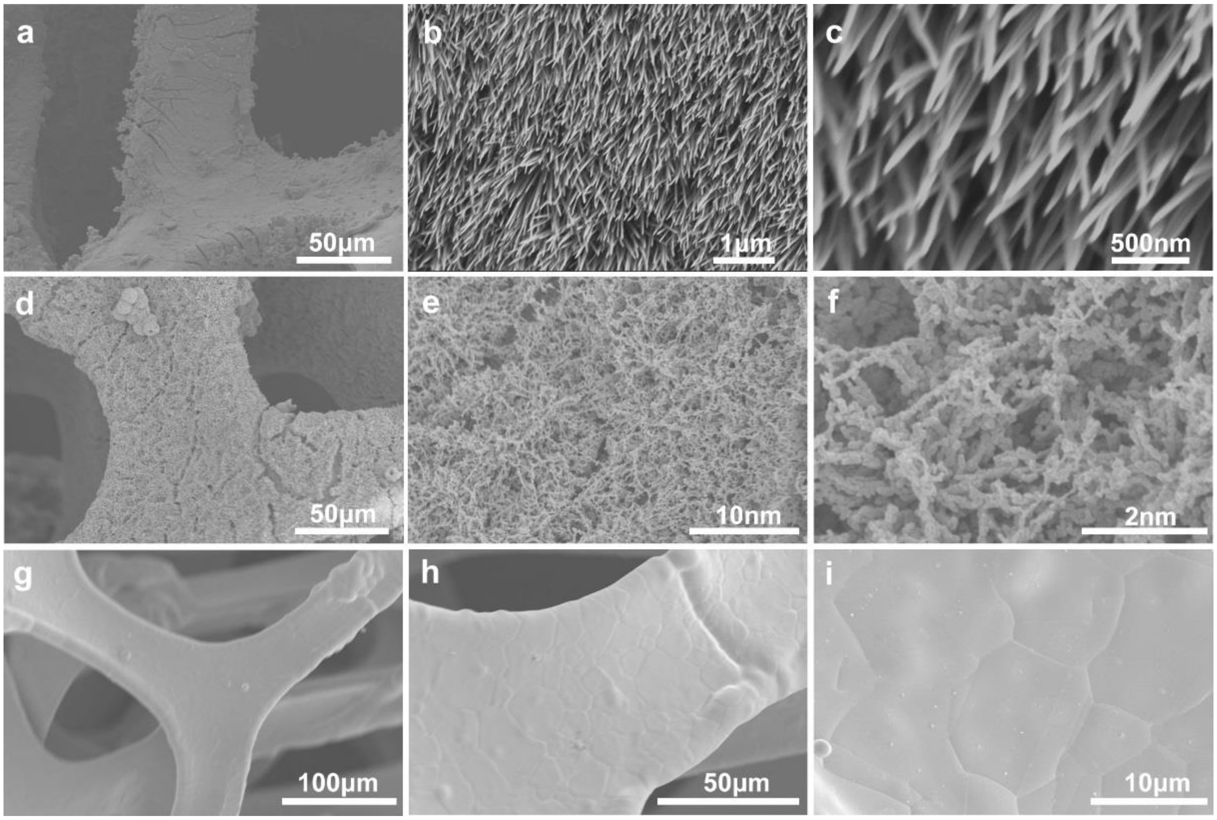

Supplementary Figure 1 | SEM images of different samples (a-c) PW-Co-precursor; (d-f) Co_3N NWA/NF; (g-i) Ni foam.

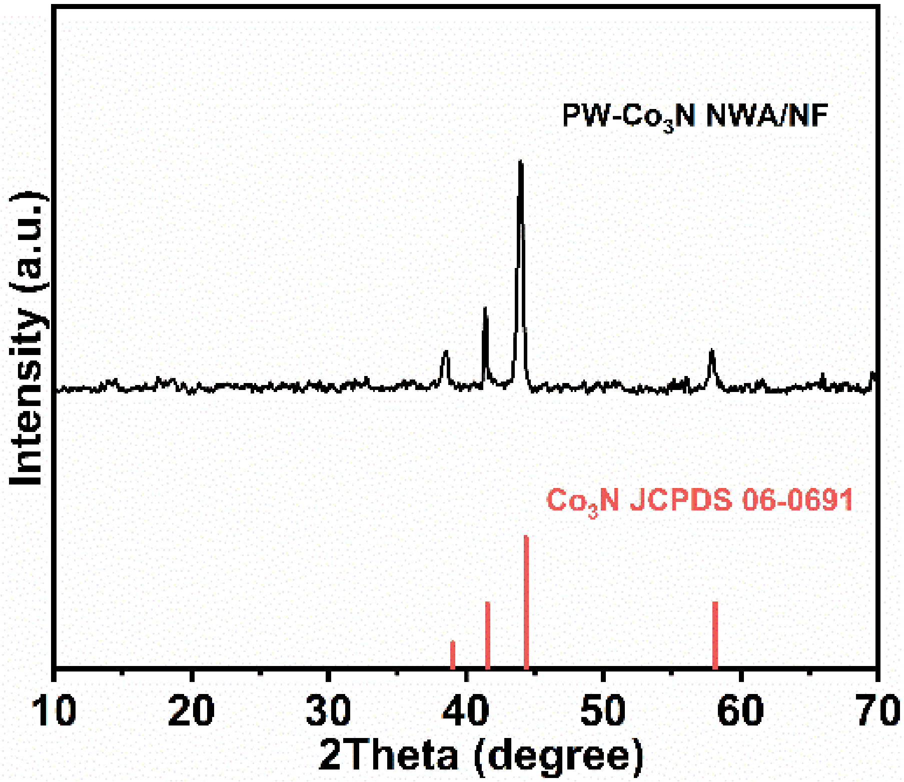

Supplementary Figure 2 | XRD pattern of PW-Co_3N NWA/NF

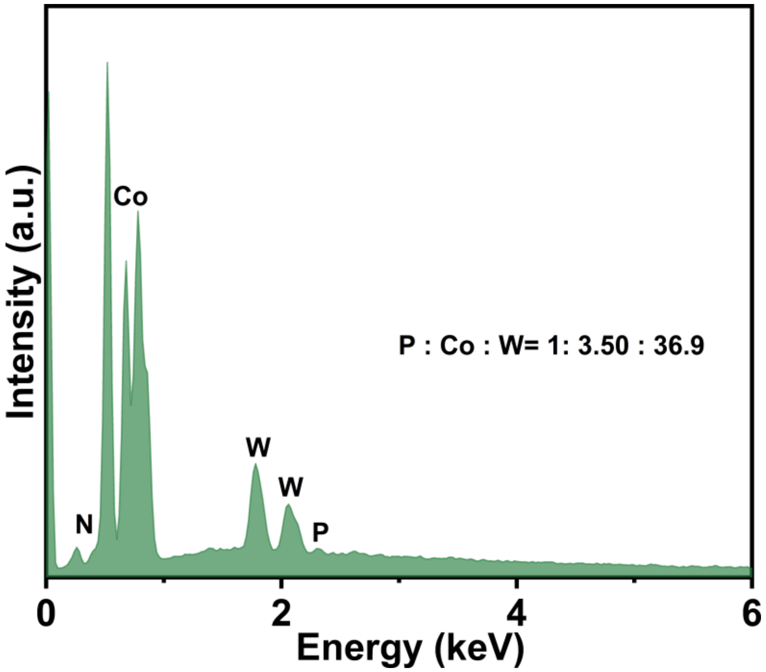

Supplementary Figure 3 | EDS spectrum of PW-Co_3N NWA/NF

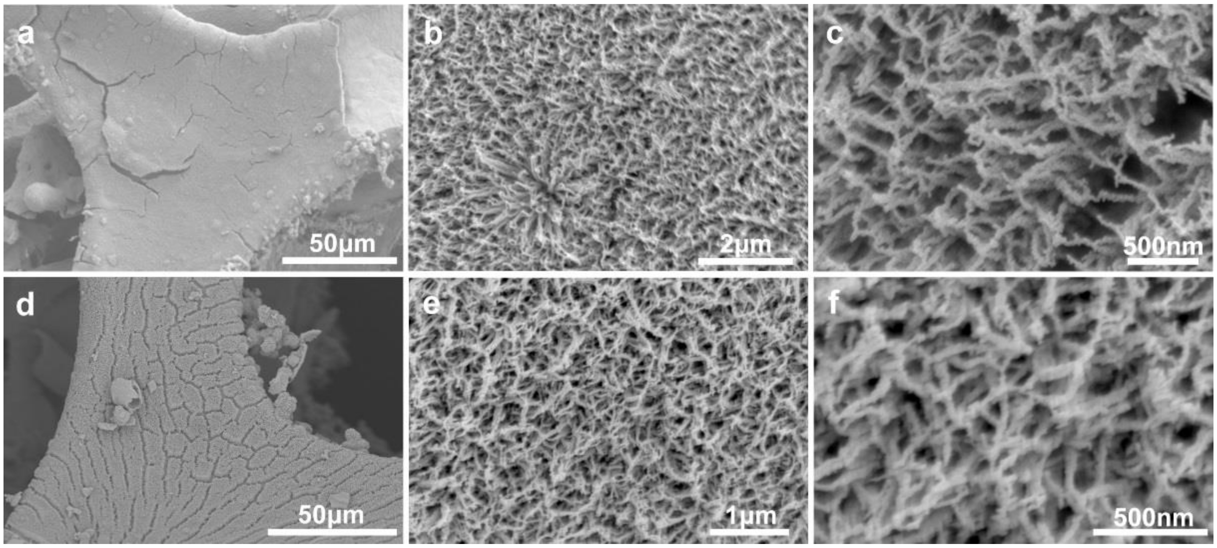

Supplementary Figure 4 | SEM images of P, W co-doped cobalt nitride nanowires arrays with different dopant ratio of PW_{12} (a) 0.004 mmoL and (b) 0.012 mmoL.

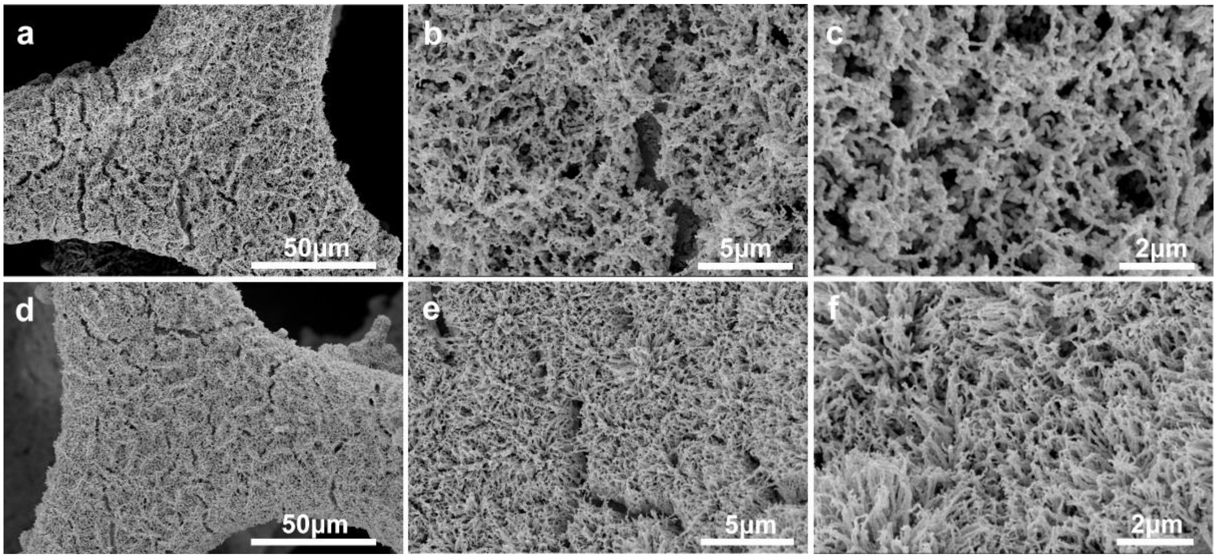

Supplementary Figure 5 | SEM images of P/W co-doped cobalt nitride nanoarrays with different annealing temperature (a-c) 350 ^{o}C and (d-f) 500 ^{o}C.

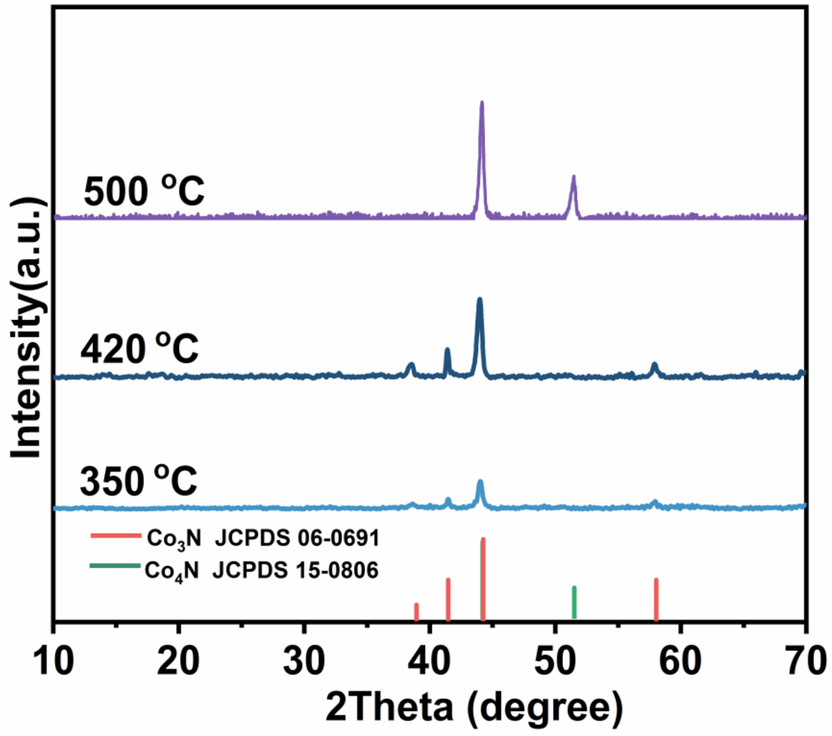

Supplementary Figure 6 | XRD pattern of P/W co-doped cobalt nitride nanoarrays with different annealing temperatures

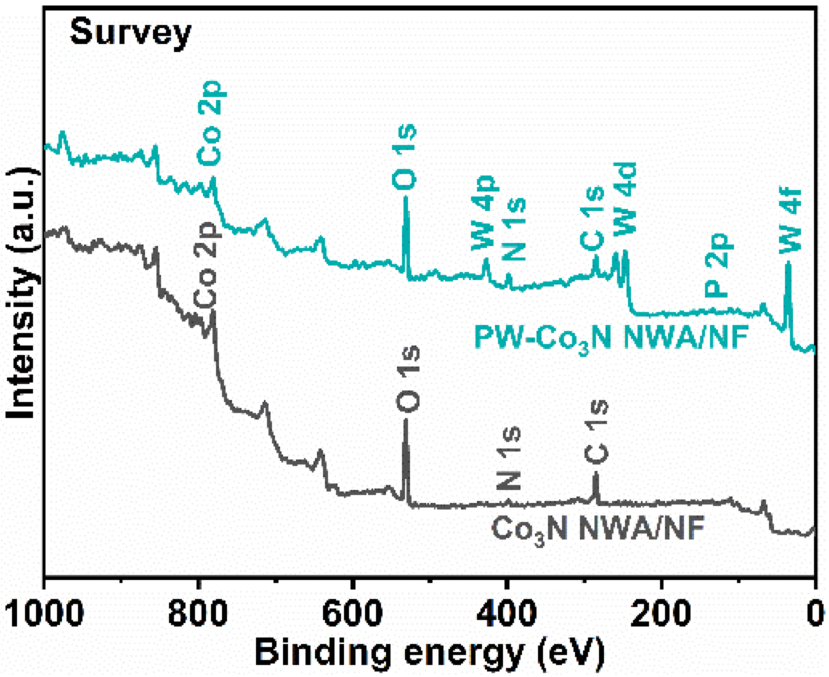

Supplementary Figure 7 | Survey XPS spectra of PW-Co_3N NWA/NF and Co_3N NWA/NF

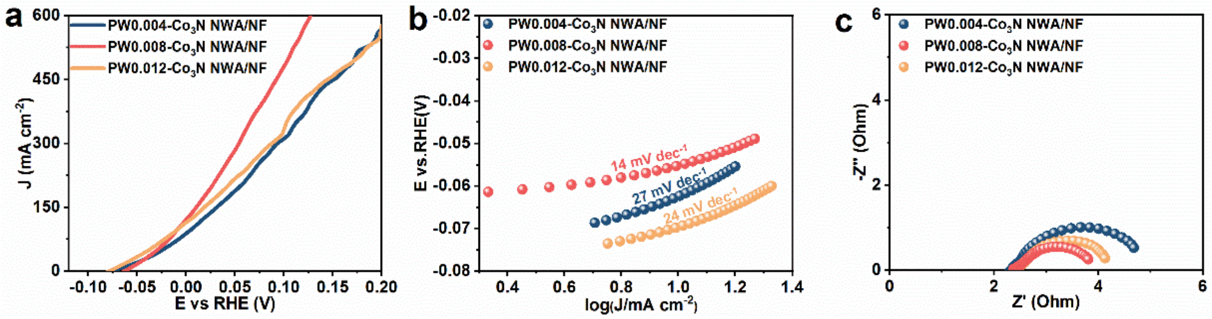

Supplementary Figure 8 | HzOR performance of PW-Co_3N NWA/NF with different dopant ratios

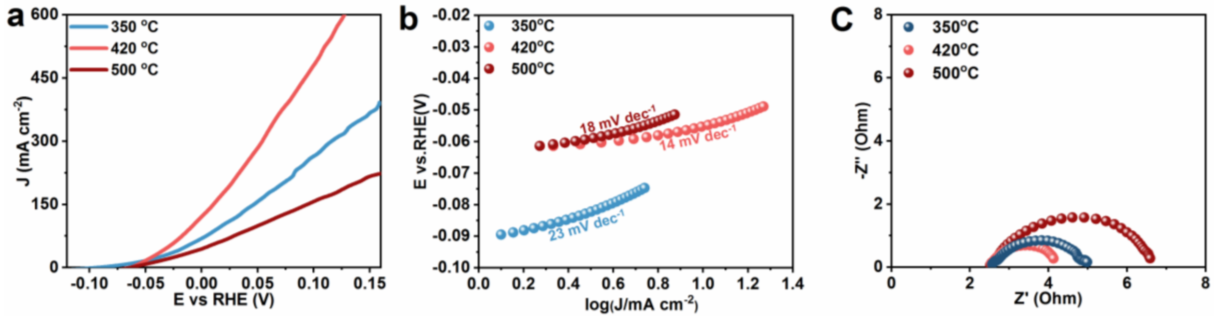

Supplementary Figure 9 | HzOR performance of PW-Co_3N NWA/NF with different annealing temperatures

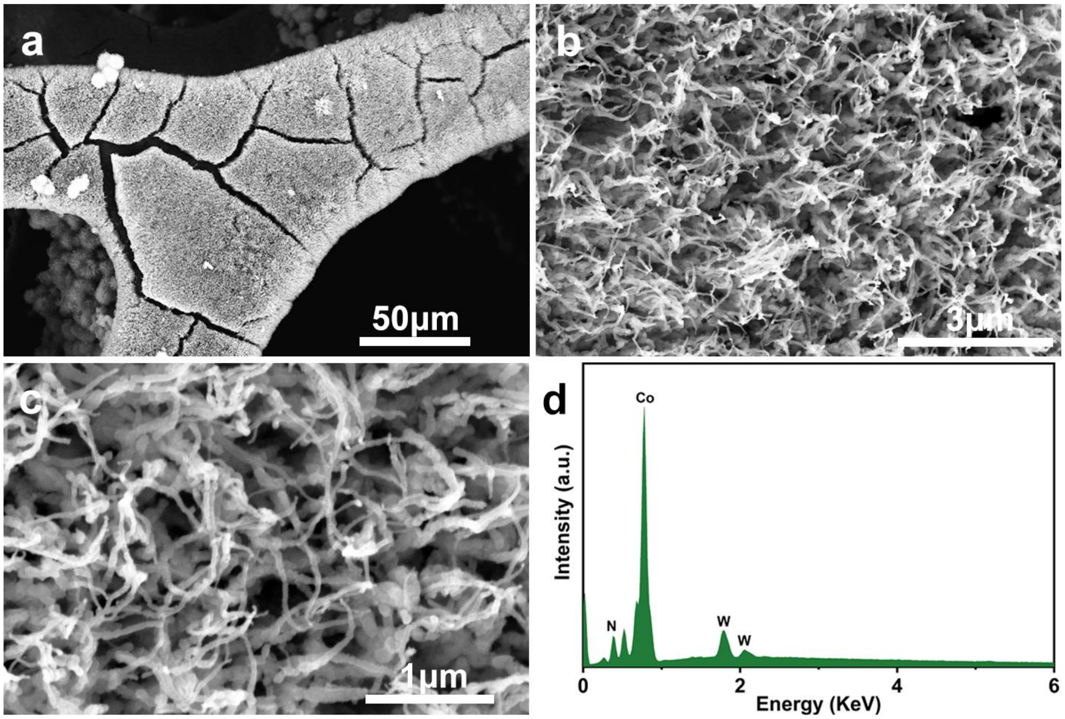

Supplementary Figure 10 | SEM images and EDS spectrum of W-Co_3N NWA/NF (a-c) SEM images with different magnifications, (d) EDS spectrum.

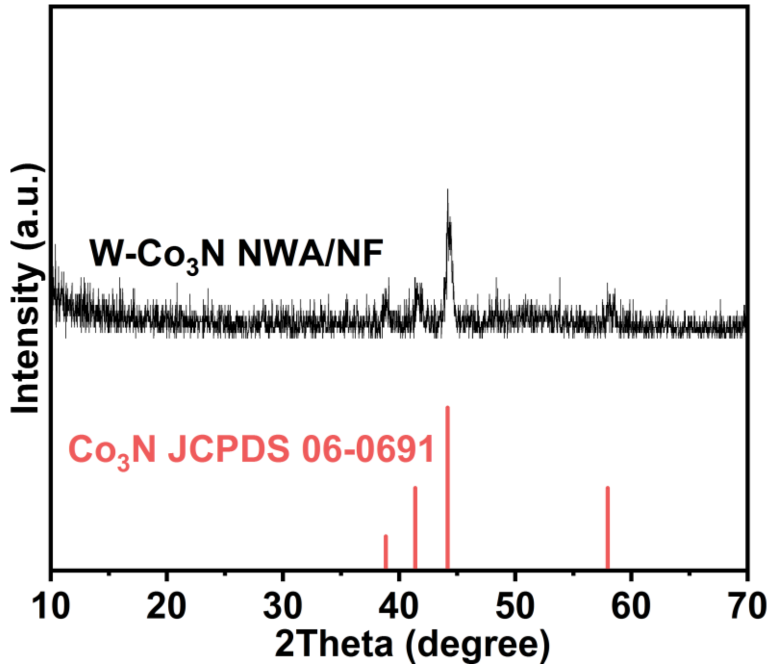

Supplementary Figure 11 | The XRD pattern of W-Co_3N NWA/NF performed on the powders scratched from Ni foam

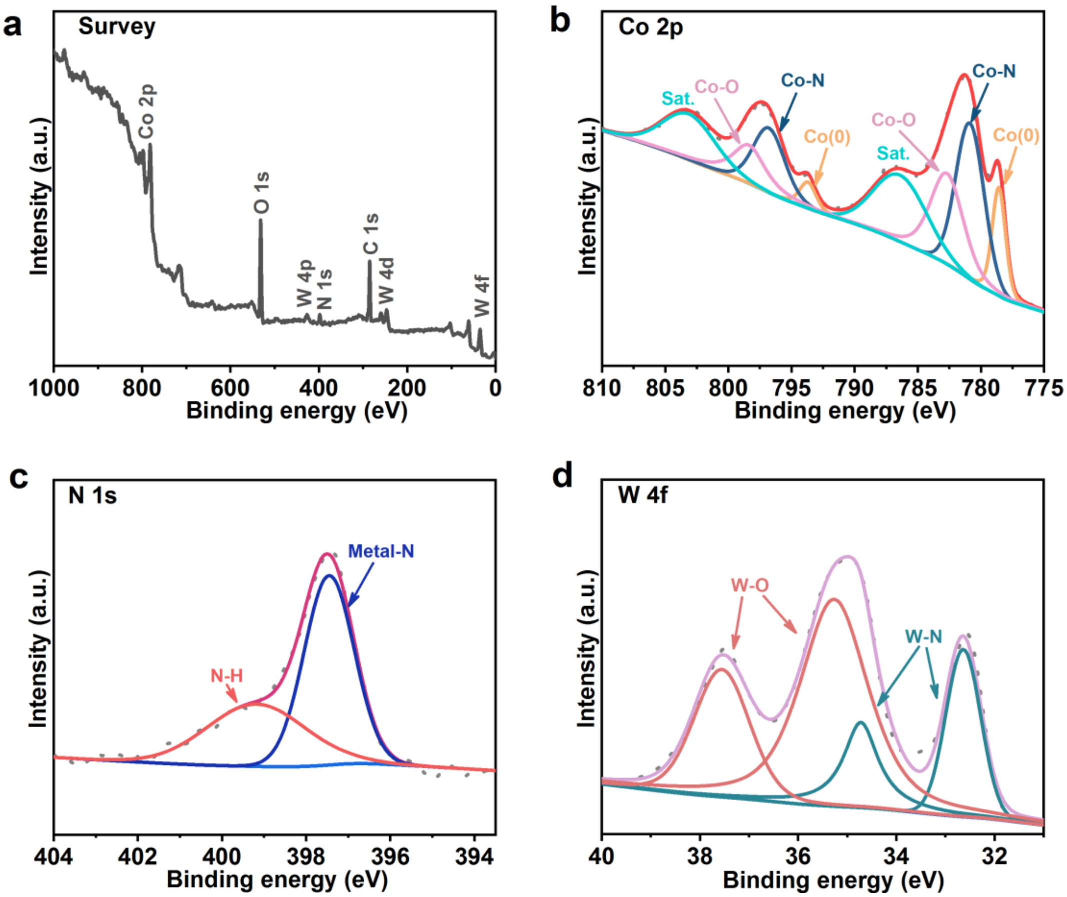

Supplementary Figure 12 | The XPS spectra of W-Co_3N NWA/NF (a) survey, high resolution spectra of (b) Co 2p, (c) N 1s, (d) W 4f.

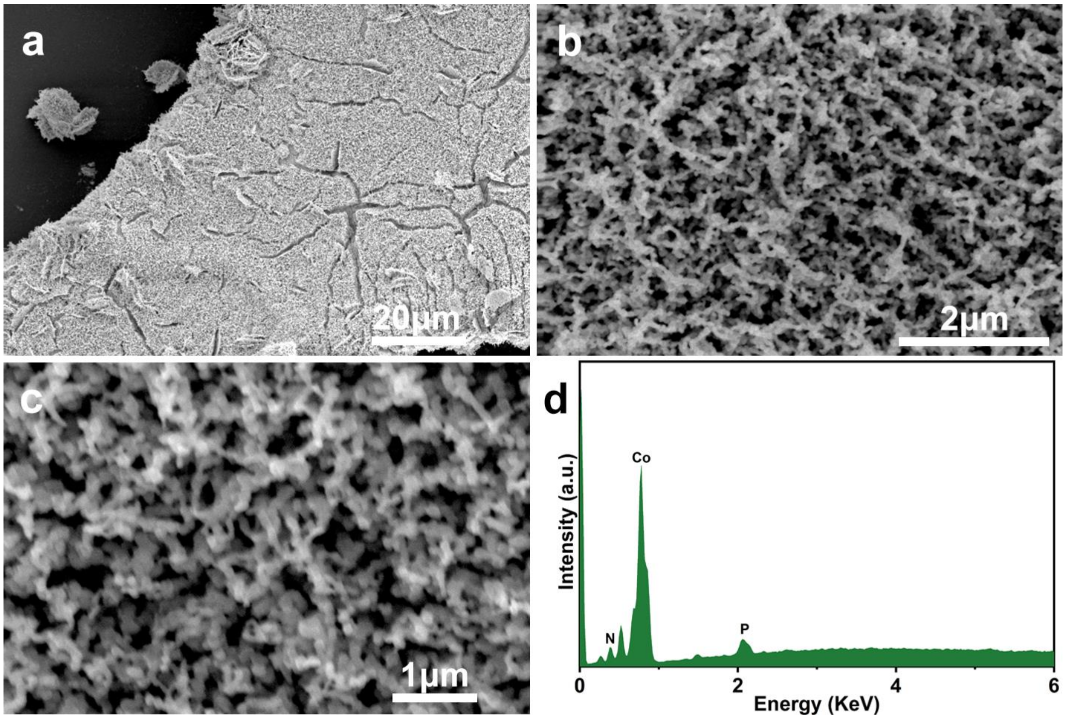

Supplementary Figure 13 | SEM images and EDS spectrum of P-Co_3N NWA/NF (a-c) SEM images, (d) EDS spectrum.

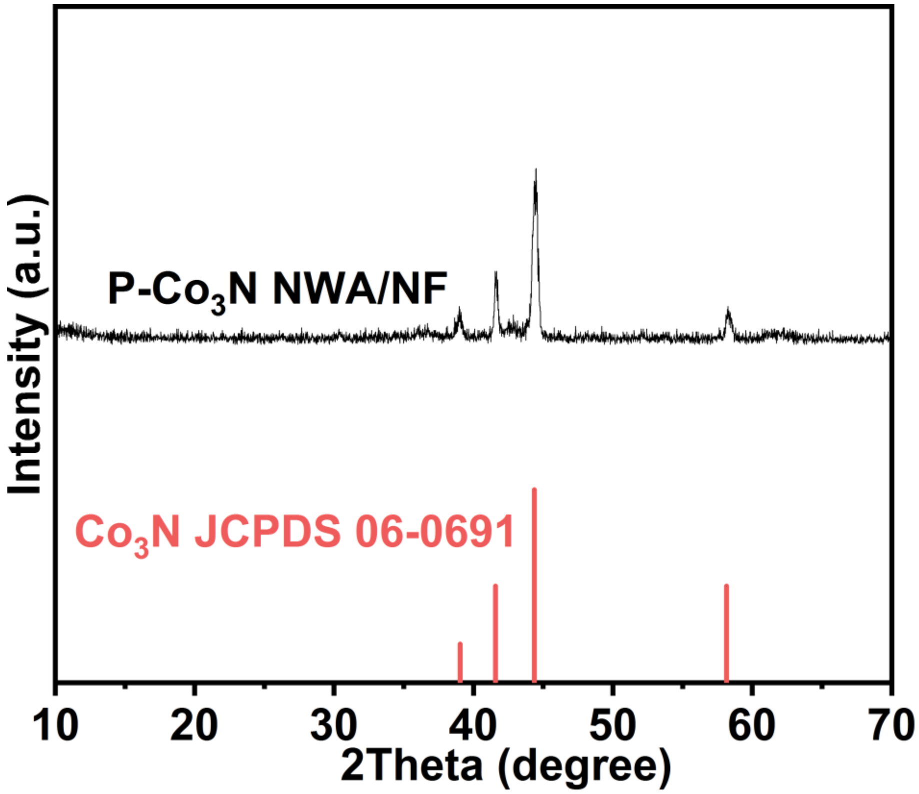

Supplementary Figure 14 | The XRD pattern of P-Co_3N NWA/NF performed on the powders scratched from Ni foam

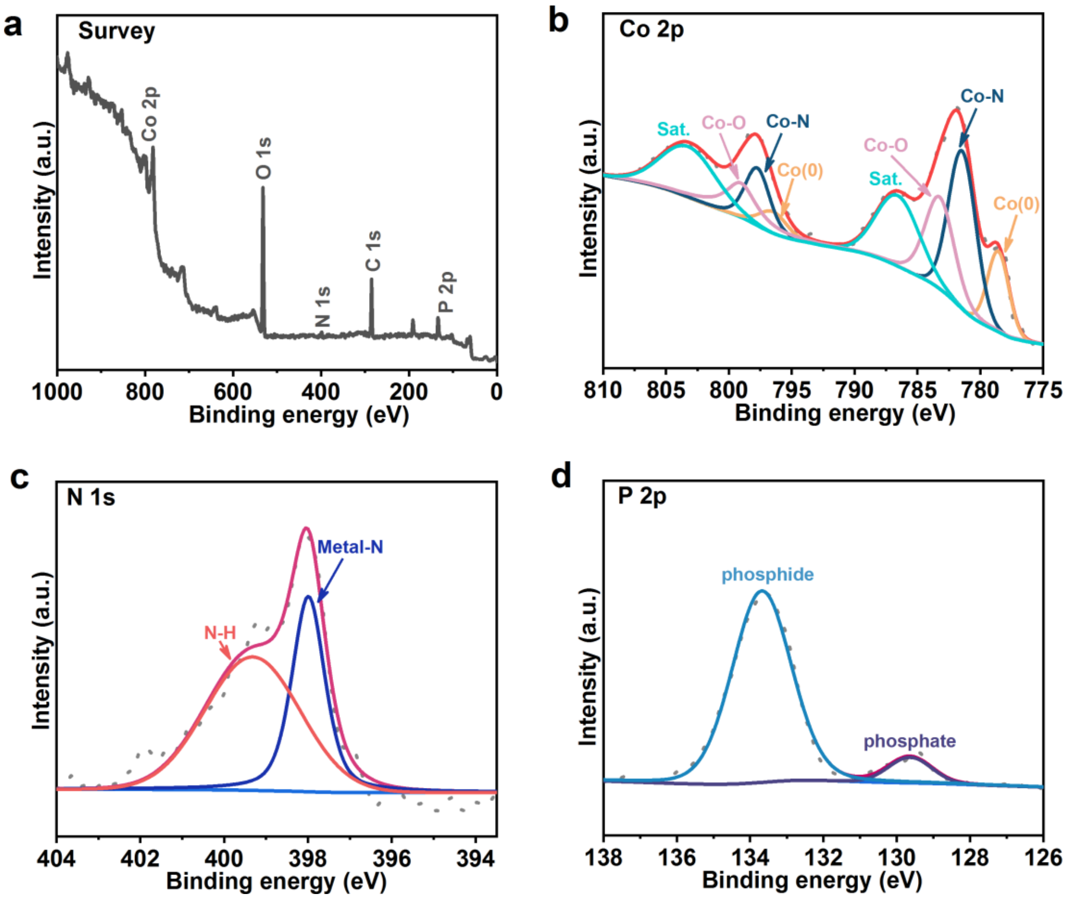

Supplementary Figure 15 | The XPS spectra of P-Co_3N NWA/NF (a) survey spectrum, high resolution spectra of (b) Co 2p, (c) N 1s, (d) P 2p.

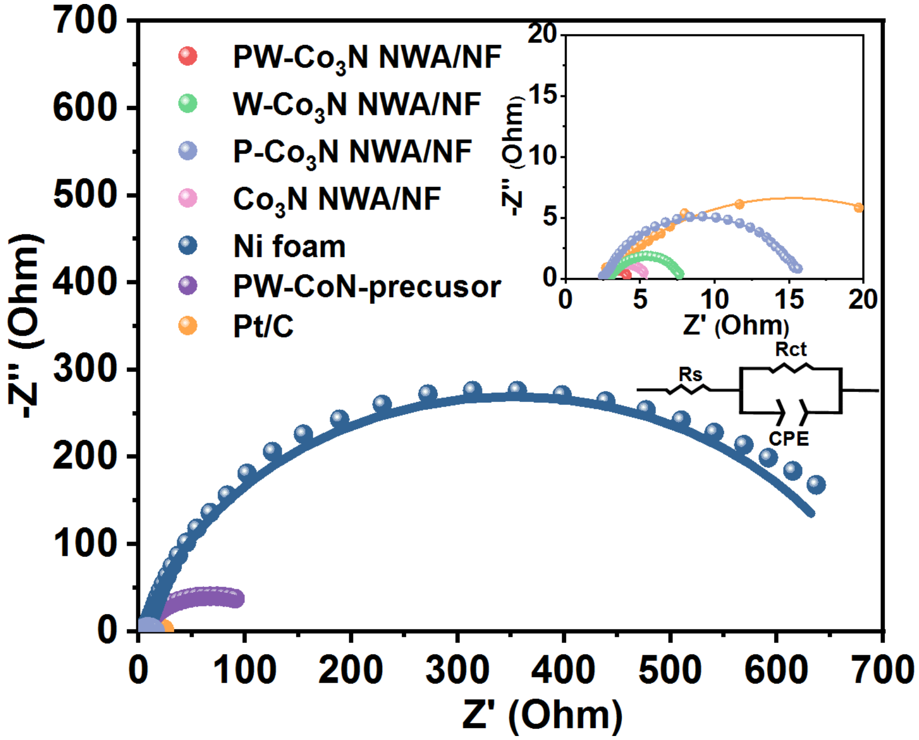

Supplementary Figure 16 | Nyquist plots of different materials for HzOR The inset is the enlarged view of Nyquist plots of PW-Co_3N NWA/NF, W-Co_3N NWA/NF, P-Co_3N NWA/NF, Co_3N NWA/NF and Pt/C.

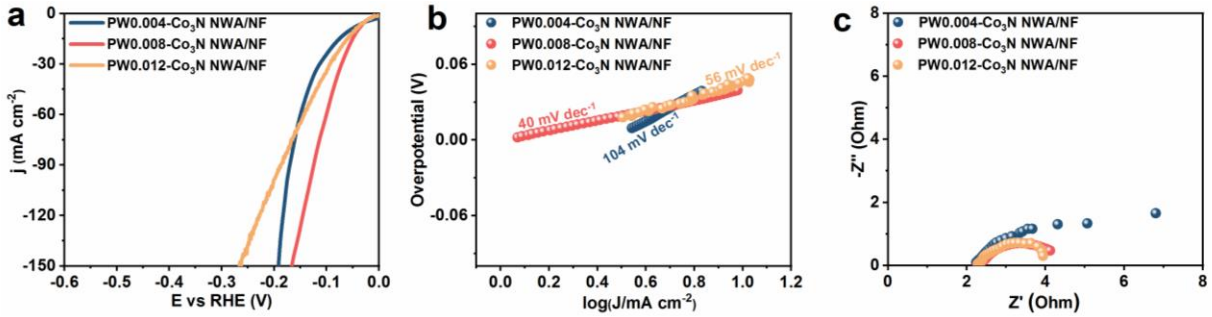

Supplementary Figure 17 | HER performance of PW-Co_3N NWA/NF with different dopant ratios

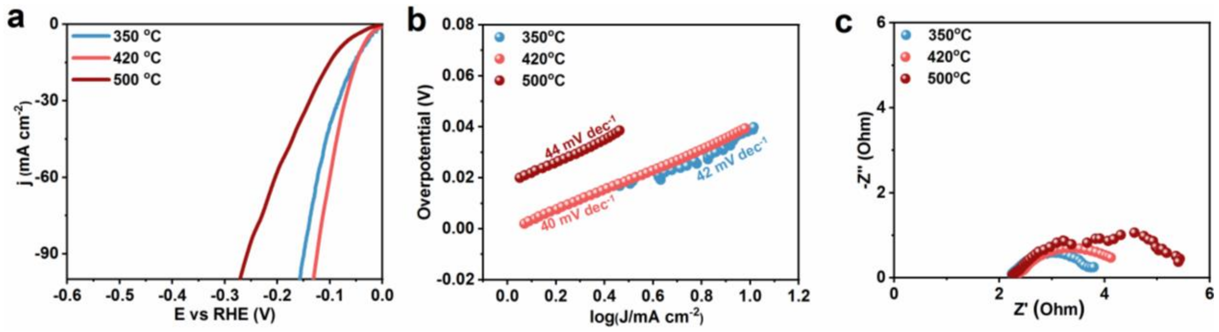

Supplementary Figure 18 | HER performance of PW-Co_3N NWA/NF with different annealing temperatures

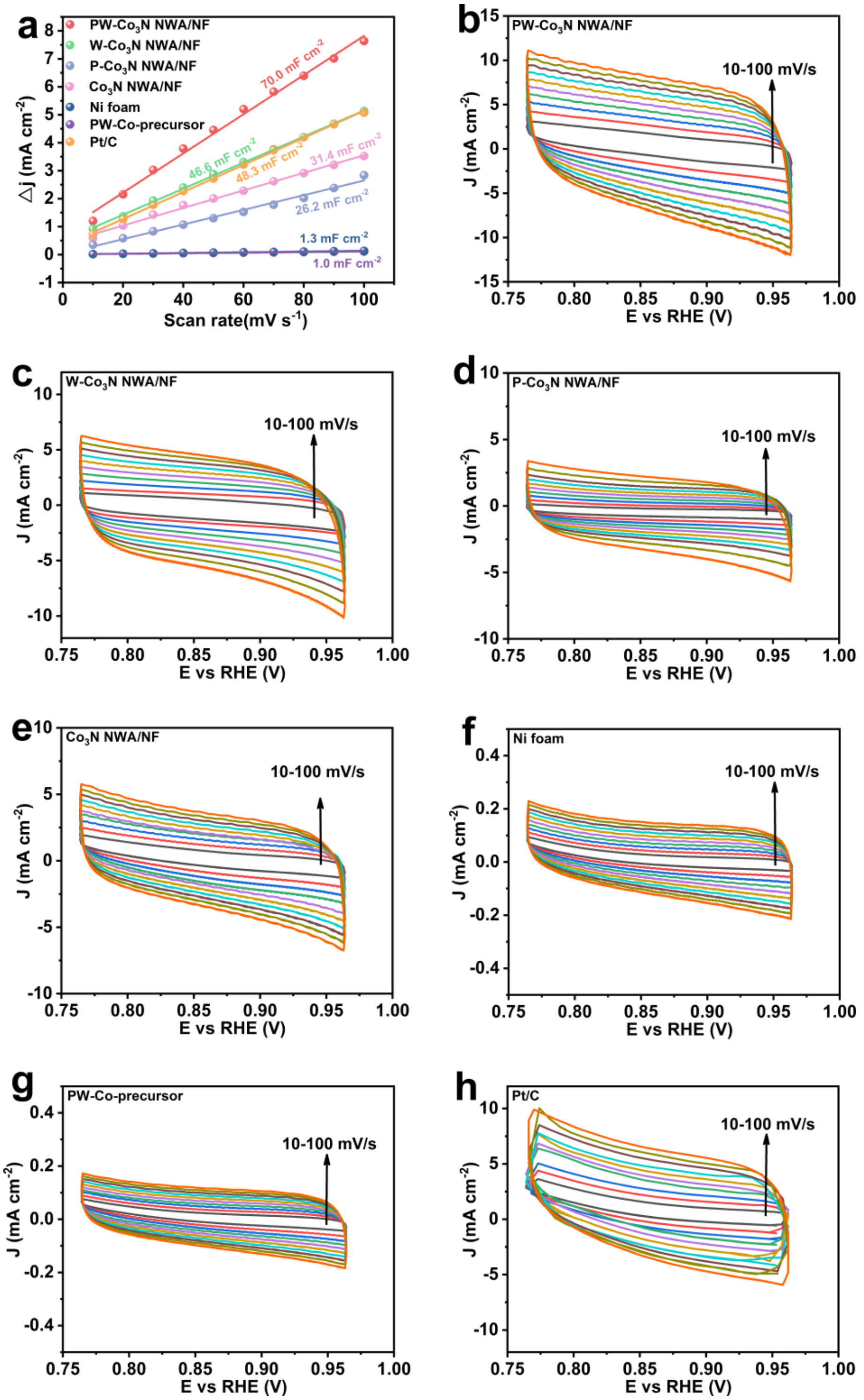

Supplementary Figure 19 | C_{dl} values and cyclic voltammogram curves (a) C_{dl} values of different materials; CV curves of (b) PW-Co_3N NWA/NF, (c) W-Co_3N NWA/NF, (d) P-Co_3N NWA/NF, (e) Co_3N NWA/NF, (f) Ni foam, (g) PW-Co-precursor and (h) Pt/C in the double layer capacitive region at the scan rates from 10 mV to 100 mV s^{-1}.

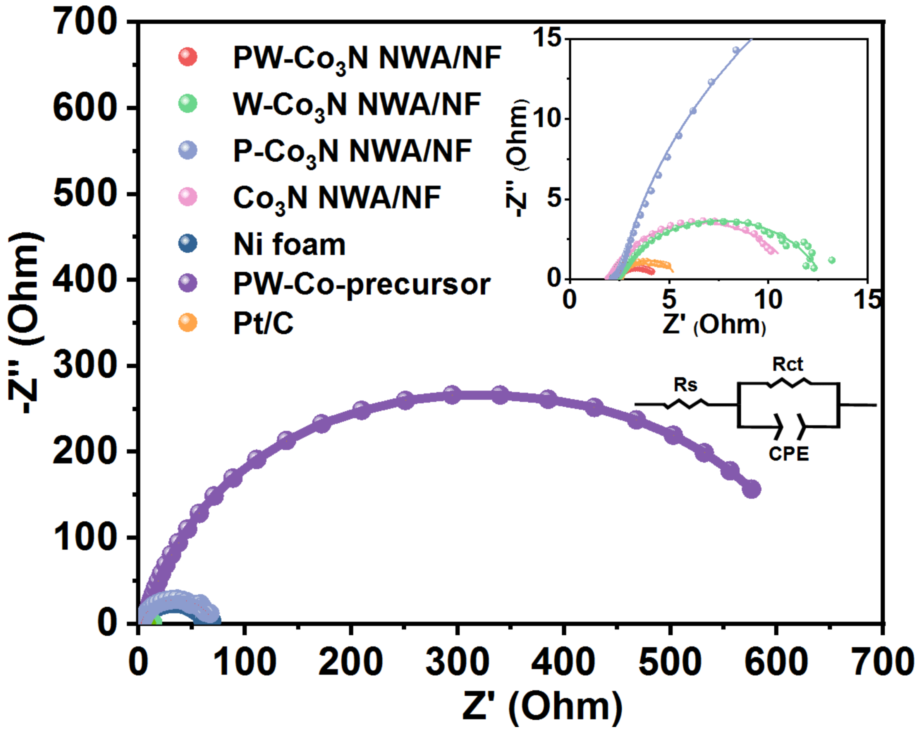

Supplementary Figure 20 | Nyquist plots of different materials for HER The inset is the enlarged view of Nyquist plots of PW-Co_3N NWA/NF, W-Co_3N NWA/NF, P-Co_3N NWA/NF, Co_3N NWA/NF, Ni foam, PW-Co-precursor and Pt/C.

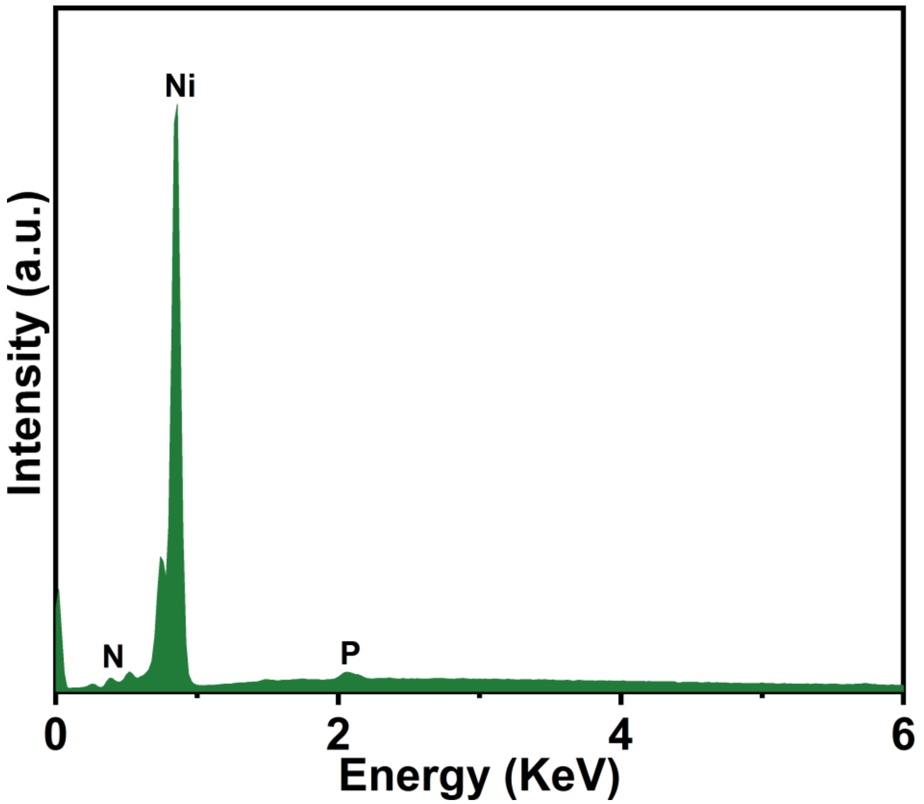

Supplementary Figure 21 | EDS results of PN/NF

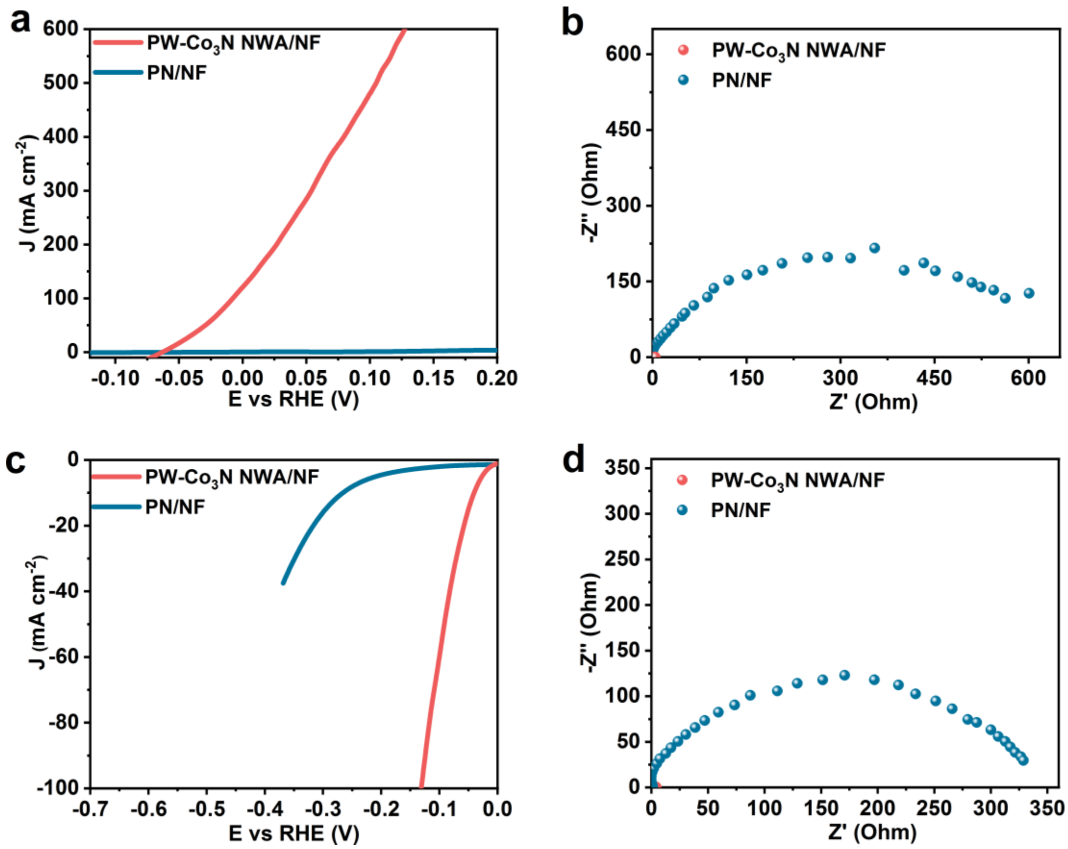

Supplementary Figure 22 | HzOR and HER performance of PN/NF and PW-Co_3N NWA/NF

HzOR: (a) LSV curves, (b) Nyquist plots; HER: (c) LSV curves, (d) Nyquist plots.

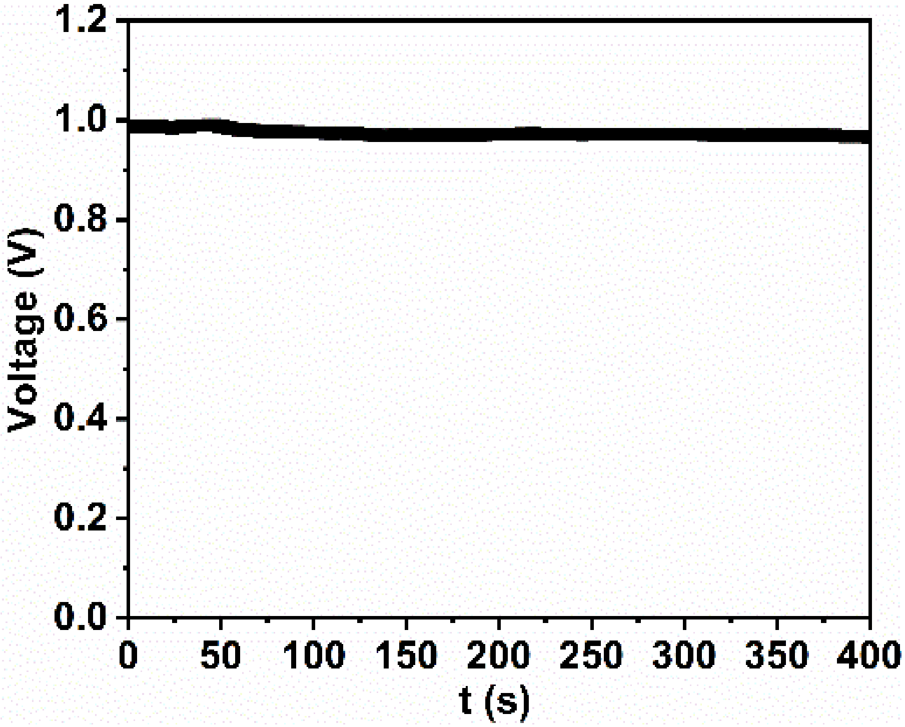

Supplementary Figure 23 | open-circuit voltage of DHzFC

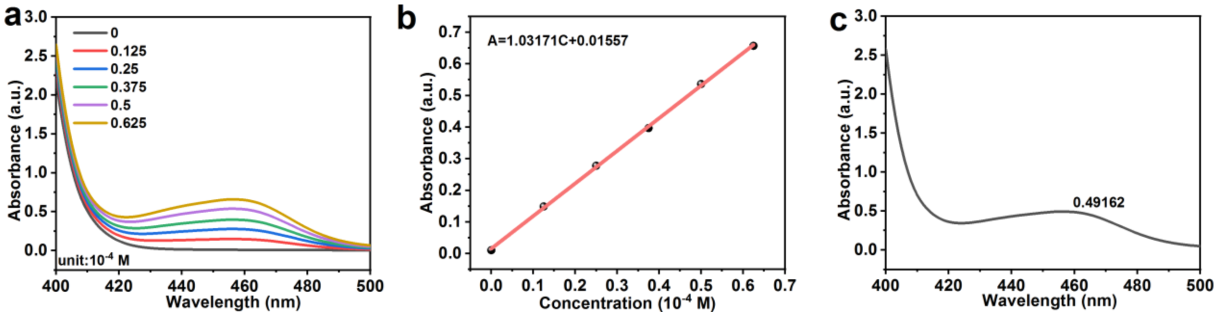

Supplementary Figure 24 | Detection of hydrazine using UV-vis spectrophotometric method (a) UV-vis absorption spectra of different concentrations of hydrazine stained with color reagent, (b) calibration curve for calculating the hydrazine concentration, (c) UV-vis absorption spectra of the diluted electrolytes from DHzFC stained with color reagent.

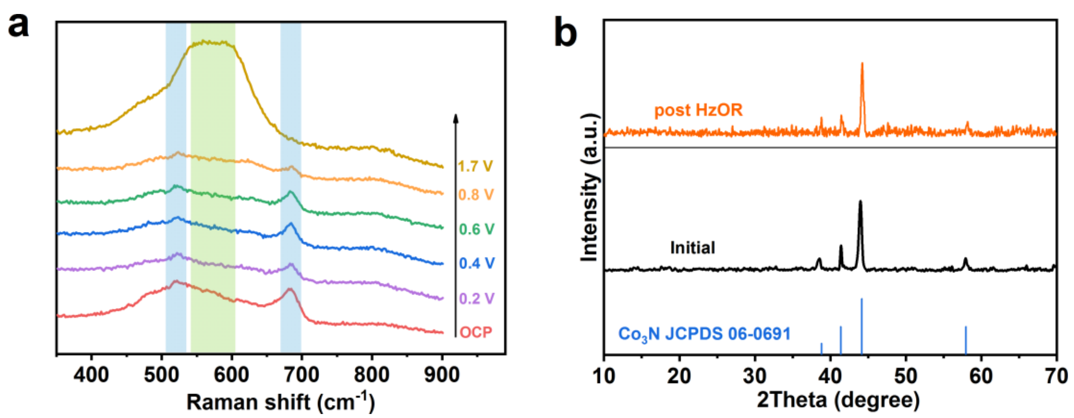

Supplementary Figure 25 | The investigation on the underlying active species for HzOR activity (a) *In-situ* potential-dependent Raman spectroscopy characterization of PW-Co_3N NWA/NF towards HzOR under programmed applied potentials in 0.1 M KOH/20 mM N_2H_4, OCP means open circuit potential; (b) XRD patterns for PW-Co_3N NWA/NF before and after long-term stability test towards HzOR.

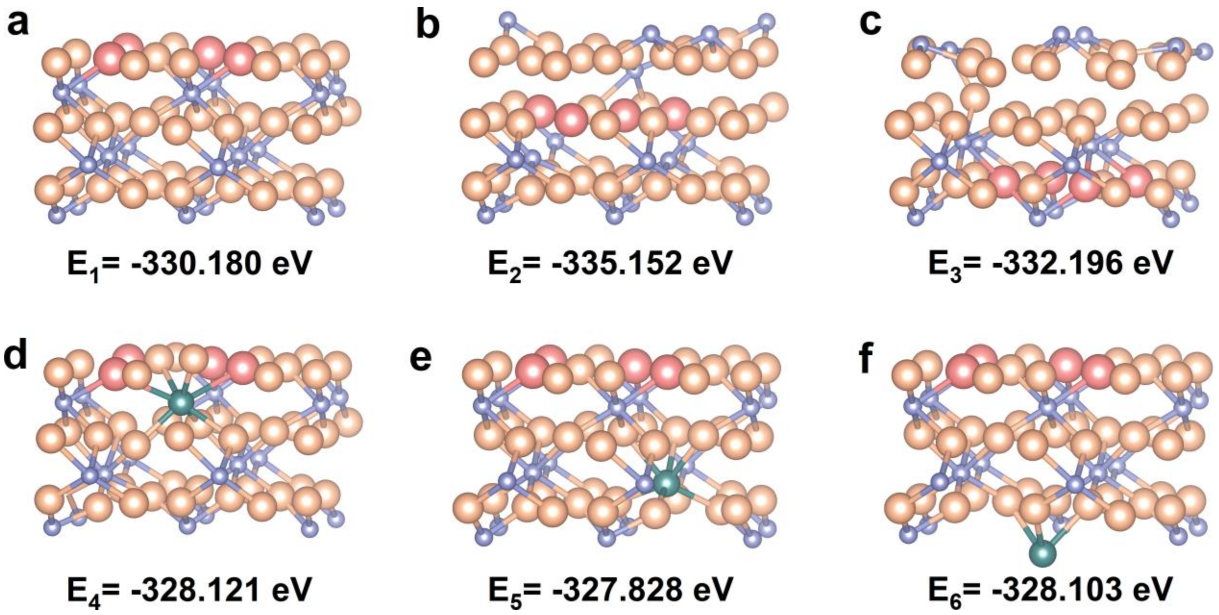

Supplementary Figure 26 | Model simulation of 4 W and 1 P atoms substituted Co_3N Model simulation of 4 W atoms substituted Co atoms at (a) first, (b) second and (c) third Co layers of Co_3N (001) surface, after geometric optimization; Model simulation of 1 P atom substituted N atom at (d) first, (e) second and (f) third N layers of sur-4 W: Co_3N surface, after geometric optimization. The balls in yellow, pink, green and purple represent Co, W, P and N atoms, respectively.

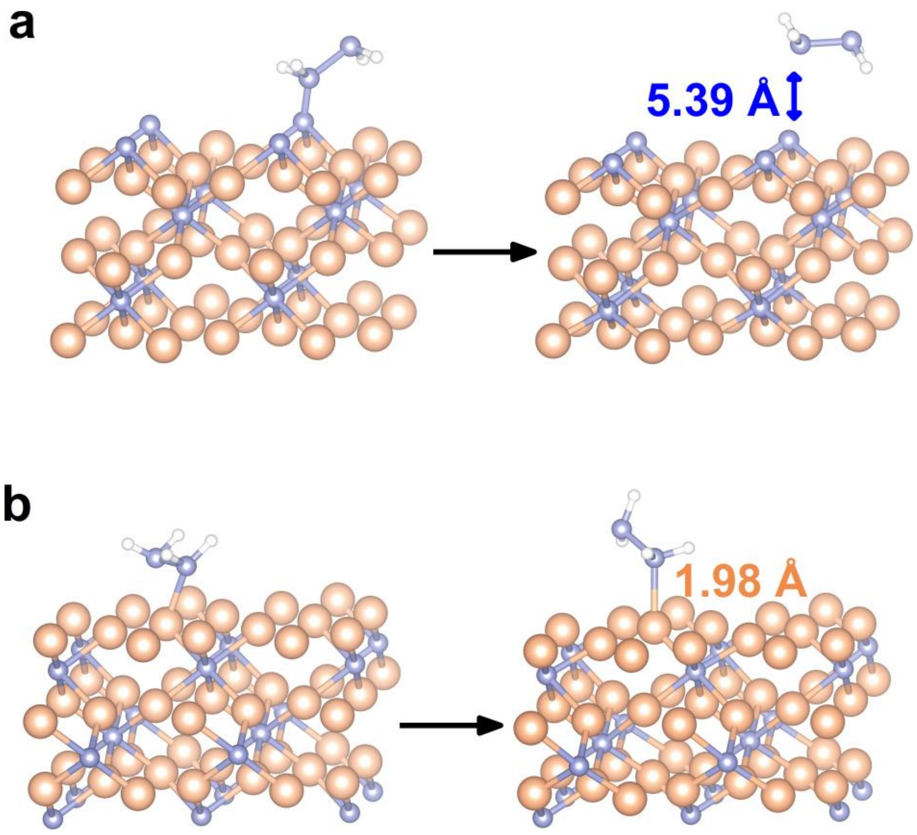

Supplementary Figure 27 | Models simulating adsorption of N_2H_4 molecule The adsorption of N_2H_4 molecule on the (a) N atom terminal and (b) Co atom terminal of Co_3N (001), respectively, before (left) and after (right) geometric optimization.

Supplementary Tables:

Supplementary Table 1. EXAFS fitting parameters at the Co K-edge and W L_3-edge for various samples. S_0^2= 0.776(Co), 0.859(W).

Sample	Shell	N^a	$R(Å)^b$	$\sigma^2(Å^2)^c$	$\Delta E_0(eV)^d$	*R* factor
**Co K-edge**						
**Co foil**	Co-Co	12	2.49	0.0063	7.2	0.0004
**CoO**	Co-O	6.0	2.06	0.0101	-3.6	0.0004
	Co-Co	12.0	2.99	0.0095		
**Co_3N**	Co-N	2.1	1.88	0.0019	-0.6	0.0007
	Co-Co	11.6	2.67	0.0073		
**PW-Co_3N**	Co-N	2.0	1.85	0.0021	-0.9	0.0065
	Co-Co	12.9	2.67	0.0119		
**W L_3-edge**						

W foil	W-W	8	2.74	0.0027	5.8	0.0040
	W-W	6	3.16	0.0027		
WO_3	W-O	4.0	1.76	0.0035	8.7	0.0082
	W-O	2.0	2.16	0.0018		
	W-O	2.1	3.22	0.0034		
	W-W	7.9	3.66	0.0062		
PW-Co_3N	W-N	1.7	1.79	0.0015	1.0	0.0015
	W-P	1.2	2.33	0.0048		
	W-Co	4.4	3,09	0.0096		
	W-Co	4.5	3.34	0.0096		

[a]*N*: coordination numbers; [b]*R*: bond distance; [c]σ^2: Debye-Waller factors; [d] ΔE_0: the inner potential correction. *R* factor: goodness of fit. S_0^2 was set to 0.776 for Co and 0.859 for W, according to the experimental EXAFS fit of Co and W foil reference by fixing CN as the known crystallographic value.

Supplementary Table 2. Comparison of the electrocatalytic activities of PW-Co_3N NWA/NF with other reported materials for HzOR.

Materials	electrolyte	J (mA cm^{-2})	Potential (mV)	Reference
**PW-Co_3N NWA/NF**	1.0 M KOH+0.1 M N_2H_4	10	-55	This work
		50	-29	
		200	27	
**Co_3Ta/C**	3.0 M KOH+0.5 M N_2H_4	25.2	60	1
**Cu_1Ni_2-N**	1.0 M KOH+0.5 M N_2H_4	10	0.5	2
**Ni_xP/Ni foam**	1.0 M NaOH+0.1 M N_2H_4	172	100	3
**Rh/N-CBs**	1.0 M KOH+0.05 M N_2H_4	10	72	4
**Fe-CoS_2**	1.0 M KOH+0.1 M N_2H_4	100	129	5
**$CoSe_2$**	1.0 M KOH+0.5 M N_2H_4	10	-17	6
**Ni_3S_2/NF**	1.0 M KOH+0.2 M N_2H_4	100	415	7

**Ni_2P/Ni foam**	1.0 M KOH+0.5 M N_2H_4	50	-25	8
**Ni-NSA**	3.0 M KOH+1.0 M N_2H_4	227.6	250	9
**NiZn**	1.0 M KOH+1.0 M N_2H_4	320	600	10

Supplementary Table 3. Comparison of the electrocatalytic activities of PW-Co_3N NWA/NF with recently reported transition metal nitride for HER in 1.0 M KOH.

Materials	η_{10} (mV)	Tafel slope (mV dec^{-1})	Reference
**PW-Co_3N NWA/NF**	41	40	This work
**NiMoN/NF**	56	45.6	11
**NiCoN/C nanocages**	103	-	12
**Ni_3N/C**	64	48	13
**Co_2N/Co/CF**	12	41.6	14
**V-Co_4N/NF**	37	44	15
**Co-Ni_3N**	194	156	16

Supplementary Table 4. Comparison of OHzS performance of PW-Co_3N NWA/NF with other work.

Materials	electrolyte	J (mA cm^{-2})	cell voltage (mV)	Reference
**PW-Co_3N NWA/NF**	1.0 M KOH+0.1 M N_2H_4	10	25	This work
		200	277	
**Cu_1Ni_2-N**	1.0 M KOH+0.5 M N_2H_4	10	240	2
**Fe-CoS_2**	1.0 M KOH+0.1 M N_2H_4	100	610	5
**$CoSe_2$**	1.0 M KOH+0.5 M N_2H_4	10	164	6
**Ni(Cu)**	1.0 M KOH+0.5 M N_2H_4	200	641	17
**Ni_2P/NF**	1.0 M KOH+0.5 M N_2H_4	100	450	8

Supplementary Table 5. Comparison of DHzFC performance of PW-Co_3N NWA/NF with other work.

Materials	Anodic fuel	temperatures	Pmax (mW cm^{-2})	OCV (V)	Reference
**PW-Co_3N NWA/NF**	1.0 M KOH+0.5 M N_2H_4	room temperature	46.3	0.98	This work
**nanostructured Cu film**	4.0 M NaOH+20 wt.% N_2H_4	room temperature	29.1	about 1	18
**NPGLs**	4.0 M NaOH+10 wt.% N_2H_4	40 °C	42.5	0.89	19
**$Pt_{53}Cu_{47}$/C**	1.0 M NaOH+1.0 M N_2H_4	60 °C	32.6	about 0.8	20
**Co-PPy/C**	1.0 M KOH+5 wt.% N_2H_4	50 °C	75	0.73	21

Supplementary Notes:

Supplementary Note 1. Calculations of total efficiency of self-power system.

The calculated efficiency can be identified by the following equation:

$$\text{Total Efficiency (TE, \%)} = N_{H_2}/2N_{DHzFC} * FE_{OHzS} * 100\ \% \qquad \textbf{(S1)}$$

Where N_{H_2} is the amount (mol) of produced hydrogen, N_{DHzFC} is the amount (mol) of the electrolyte of DHzFC and FE is the Faraday efficiency (%) of OHzS. The amount (mol) of the consumed hydrazine were carefully measured by a UV-vis spectrophotometric method proposed by Watt and Chrisp[22-24] and calculated via Faraday efficiency, respectively. The color reagent is the mixed solution of 1.0 g p-(dimethylamino) benzaldehyde, 50 mL ethanol and 5 mL of 0.12 M HCl. Meanwhile, firstly, 50 μL of electrolyte from DHzFC after reaction was added into measuring flask and DIW was also added into the same measuring flask till 500 mL in total volume. Then 1 mL of the above solution from the measuring flask was mixed with 1 mL color reagent and 4 mL of 0.12 M HCl. After standing at room temperature for 20 min, the UV-vis spectrum of the solution was collected. The concentration-absorbance curves were calibrated using standard hydrazine solution in a series of concentrations (as indicated in Supplementary Fig. 24a-b). Following this strategy, the total efficiency is calculated to be about 45.8 % in our system, which is outstanding compared to other hydrogen generation system, such as mechanical energy driven self-power system[25] (43.8%), hybrid energy cell[26] (16%) and water photolysis[27] (12.3%).

Supplementary Note 2. Model optimization of 4 W and 1 P atoms substituted Co_3N.

We calculated 4 W atoms substituted for 4 Co atoms in different Co layer of Co_3N (001) surface. After geometric optimization, as shown in Supplementary Fig. 26a, total energy of 4 W substituted for 4 Co atom at first Co layers of Co_3N (001) surface are -330.180 eV, and its structure shows no significant distortion. Contrastively, there will be serious structural distortions for the system with 4 W doped at second and third Co layers of Co_3N (001) planes where the N atoms of second layer totally deviate from its original position, as shown in Supplementary Fig. 26b-c. This is not reasonable since the crystal structure can be well maintained after W doping according to the XRD results, although the total energy (-335.152 eV for doping second Co layer, -332.196 eV for doping third Co layer) are lower compared to the system with surface doping model. Therefore, 4 W substituted for 4 Co atom at first Co layers of Co_3N (001) surface (denoted as sur-4 W: Co_3N) is the most favorable configuration than that of the other Co layers. Secondly, we calculated the substitution doping of a single P atom in different N layer of sur-4 W: Co_3N surface. After geometric optimization, as shown in Fig. 26d-f, the total energy of P substituted for N atom at first, second and third N layers of sur-4 W: Co_3N surface are calculated to be -328.121 eV, -327.828 eV, and -328.103 eV, respectively. Their structures show no significant distortion which are consistent with the XRD results after P/W doping. Thus the results indicate that the P is favorable to replace N atom at first N layers of sur-4 W: Co_3N surface due to the lowest energy configuration. Based on these results, we adopt the optimal structure of replace surface Co atoms in Co_3N (001) with W atoms and subsurface N atom with P atom model during our calculation.

Supplementary References:

1. Feng, G. et al. Atomically ordered non-precious Co_3Ta intermetallic nanoparticles as high-performance catalysts for hydrazine electrooxidation. *Nat. Commun.* **10**, 4514 (2019).

2. Wang, Z. et al. Copper–nickel nitride nanosheets as efficient bifunctional catalysts for hydrazine-assisted electrolytic hydrogen production. *Adv. Energy Mater.* **9**, 1900390 (2019).

3. Wen, H. et al. In situ grown Ni phosphide nanowire array on Ni foam as a high-performance catalyst for hydrazine electrooxidation. *Appl. Catal. B: Environ.* **241**, 292-298 (2019).

4. Jia, N. et al. 0.2 V electrolysis voltage-driven alkaline hydrogen production with nitrogen-doped carbon nanobowl-supported ultrafine Rh nanoparticles of 1.4 nm. *ACS Appl. Mater. Interfaces* **11**, 35039-35049 (2019).

5. Liu, X. et al. Self-powered H_2 production with bifunctional hydrazine as sole consumable. *Nat. Commun.* **9**, 4365 (2018).

6. Zhang, J.-Y. et al. Anodic hydrazine oxidation assists energy-efficient hydrogen evolution over a bifunctional cobalt perselenide nanosheet electrode. *Angew. Chem. Int. Ed.* **57**, 7649-7653 (2018).

7. Liu, G. et al. Vapor-phase hydrothermal transformation of a nanosheet array structure $Ni(OH)_2$ into ultrathin Ni_3S_2 nanosheets on nickel foam for high-efficiency overall water splitting. *J. Mater. Chem. A* **6**, 19201-19209 (2018).

8. Tang, C. et al. Energy-saving electrolytic hydrogen generation: Ni_2P nanoarray as a high-performance non-noble-metal electrocatalyst. *Angew. Chem. Int. Ed.* **56**, 842-846 (2017).

9. Kuang, Y., Feng, G., Li, P., Bi, Y., Li, Y. & Sun, X. Single-crystalline ultrathin nickel nanosheets array from in situ topotactic reduction for active and stable electrocatalysis. *Angew. Chem. Int. Ed.* **55**, 693-697 (2016).

10. Serov, A. et al. Anode catalysts for direct hydrazine fuel cells: from laboratory test to an electric vehicle. *Angew. Chem. Int. Ed.* **53**, 10336-10339 (2014).

11. Yu, L. et al. Non-noble metal-nitride based electrocatalysts for high-performance alkaline seawater electrolysis. *Nat. Commun.* **10**, 5106 (2019).

12. Lai, J., Huang, B., Chao, Y., Chen, X. & Guo, S. Strongly coupled nickel–cobalt nitrides/carbon hybrid nanocages with Pt-like activity for hydrogen evolution catalysis. *Adv. Mater.* **31**, 1805541 (2019).

13. Ni, W., Krammer, A., Hsu, C.-S., Chen, H. M., Schüler, A. & Hu, X. Ni_3N as an active hydrogen oxidation reaction catalyst in alkaline medium. *Angew. Chem. Int. Ed.* **58**, 1-6 (2019).

14. Song, F. et al. Interfacial Sites between cobalt nitride and cobalt act as bifunctional catalysts for hydrogen electrochemistry. *ACS Energy Lett.*, 1594-1601 (2019).

15. Chen, Z. et al. Tailoring the d-band centers enables Co_4N nanosheets to be highly active for hydrogen evolution catalysis. *Angew. Chem. Int. Ed.* **57**, 5076-5080 (2018).

16. Zhu, C. et al. In situ grown epitaxial heterojunction exhibits high-performance electrocatalytic water splitting. *Adv. Mater.* **30**, 1705516 (2018).

17. Sun, Q. et al. Bifunctional copper-doped nickel catalysts enable energy-efficient hydrogen production via hydrazine oxidation and hydrogen evolution reduction. *ACS Sustainable Chem. Eng.* **6**, 12746-12754 (2018).

18. Lu, Z. et al. Superaerophobic electrodes for direct hydrazine fuel cells. *Adv. Mater.* **27**, 2361-2366 (2015).

19. Yan, X., Meng, F., Xie, Y., Liu, J. & Ding, Y. Direct N_2H_4/H_2O_2 fuel cells powered by nanoporous gold leaves. *Sci. Rep.* **2**, 941 (2012).

20. Crisafulli, R. et al. On the promotional effect of Cu on Pt for hydrazine electrooxidation in alkaline medium. *Appl. Catal. B: Environ.* **236**, 36-44 (2018).

21. Yoshimura, K. et al. Imidazolium cation based anion-conducting electrolyte membranes prepared by radiation induced grafting for direct hydrazine hydrate fuel cells. *J. Electrochem. Soc.* **161**, F889-F893 (2014).

22. Watt, G. W. & Chrisp, J. D. Spectrophotometric method for determination of hydrazine. *Anal. Chem.* **24**, 2006-2008 (1952).

23. He, C. et al. Identification of FeN_4 as an efficient active site for electrochemical N_2 reduction. *ACS Catal.* **9**, 7311-7317 (2019).

24. Jin, H. et al. Nitrogen vacancies on 2D layered W_2N_3: a stable and efficient active site for nitrogen reduction reaction. *Adv. Mater.* **31**, 1902709 (2019).

25. Tang, W., Han, Y., Han, C. B., Gao, C. Z., Cao, X. & Wang, Z. L. Self-powered water splitting using flowing kinetic energy. *Adv. Mater.* **27**, 272-276 (2015).

26. Yang, Y. et al. A hybrid energy cell for self-powered water splitting. *Energy Environ. Sci.* **6**, 2429-2434

(2013).

27. Luo, J. et al. Water photolysis at 12.3% efficiency via perovskite photovoltaics and earth-abundant catalysts. *Science* **345**, 1593-1596 (2014).
